# Towards positive net outcomes for biodiversity, and developing safeguards to accompany headline biodiversity indicators

**DOI:** 10.1038/s44185-025-00095-5

**Published:** 2025-08-11

**Authors:** J. W. Bull, I. Taylor, A. de Valença, R. IJspeert, B. van Erve, P. Modernel, J. A. C. Poore

**Affiliations:** 1 Department of Biology, University of Oxford, Oxford, UK; 2 Wild Business Ltd, London, UK; 3 Metabolic, Amsterdam, Netherlands; 4 Duurzame Zuivelketen, The Hague, Netherlands; 5 FrieslandCampina, Amersfoort, Netherlands

**Keywords:** Environmental impact, Biodiversity, Conservation biology

## Abstract

Achieving the Global Biodiversity Framework will necessitate whole production systems contributing towards ‘halting and reversing’ net biodiversity loss, counterbalancing negative impacts with comparable gains. Here, we report on an illustrative quantitative exploration into the feasibility of monitoring for positive net biodiversity outcomes for the Dutch dairy production sector, using a composite metric. We analysed performance data from 8,950 dairy farms across the Netherlands, combining these data into an integrated biodiversity index. Usefully, this index allowed us to calculate sectoral baseline biodiversity impacts, and explore possible biodiversity strategies. We show that the largest overall source of impacts is imported feed; interestingly, nutrient loads contribute little to the footprint, despite representing an important political issue nationally. This highlights a general risk in using single indices to track net biodiversity outcomes: that they could result in an exclusionary focus, and perverse outcomes. Consequently, we develop safeguards to accompany the index; showing the necessity of incorporating safeguards, but also that meeting them could reduce sectoral biodiversity impacts by ~94%. Our proposed strategies vary in feasibility, all requiring trade-offs between biodiversity, land availability, and production.

## Introduction

In late 2022, the Convention on Biological Diversity adopted the Global Biodiversity Framework, aiming to “halt and reverse biodiversity loss to put nature on a path to recovery”^1^. Achieving this mission requires addressing biodiversity impacts not only from direct economic activities but also those embedded in global supply chains. Agriculture, as a leading driver of biodiversity loss, presents both challenges and opportunities in this context^2,3^. The path to global nature recovery will involve combining reactive mitigation for negative impacts on biodiversity with proactive biodiversity conservation and restoration initiatives^4^. Key principles will include that biodiversity impacts caused by economic development are quantified, reduced where possible, otherwise compensated for through biodiversity offsets, and accompanied by additional proactive conservation initiatives; leading to net positive biodiversity impacts overall. Here, we explore ‘net outcome’ approaches on biodiversity at the scale of a national agricultural production system (the Dutch dairy sector, using data collected via the established Dutch ‘Biodiversity Monitor’), as one necessary step in quantifying pathways towards global biodiversity goals.

The idea of ‘net outcome’ type mechanisms for biodiversity (i.e. those that seek to quantify biodiversity losses and sum them against comparably quantified gains, to calculate a net outcome for biodiversity) is not new, and currently forms the basis of established or emerging policy in over 100 countries worldwide, a growing conservation portfolio, as well as underpinning environmental markets worth billions of dollars a year^5^. Many of the theoretical challenges associated with achieving neutral or even positive net outcomes have been explored in the scientific literature—with the most acute current barriers more related to practical considerations, e.g. monitoring, implementation, and demonstration in practice (e.g. refs. ^6,7^).

Crucially, in implementing the Global Biodiversity Framework, it will be insufficient to consider only the direct biodiversity impacts of economic activities, but also necessary to consider their indirect impacts on biodiversity that are embedded in the supply chain. This thinking partly underpins the emerging concept of ‘nature positive’^3,8^—a specific type of positive net outcome approach—current definitions for which are explored in ref. ^8^, but which under the definition we assume (see below and www.naturepositive.org) is directly aligned with the CBD post-2020 mission. The evaluation of biodiversity impacts for specific organisations—both direct and indirect—as a basis for developing positive net outcome-type strategies (again, of which ‘nature positive’ is one example), has started to receive attention in the recent scientific literature^9^. This includes efforts to understand the organisational impacts of food consumption^10^. Nonetheless, of all of the key sectors in the global economy, agriculture is one that has seen relatively little attention in terms of net outcomes approaches. Agriculture will be an extremely influential sector in developing net outcome pathways: fundamental to society, but a leading global cause of biodiversity loss (e.g. refs. ^11–13^), and one that will play a key role in whether international agreements on climate change can be achieved^14^. Equally, agriculture could play an important role in biodiversity recovery—not only through reduction of impacts, but through restorative practices^15^. Further, agriculture as a sector represents a clear illustrative example of the challenges in trading off any efforts to stay within physical planetary boundaries with efforts to ensure society can meet core social objectives on wellbeing^16^.

Here, we use an illustrative case study of dairy production in the Netherlands. Dairy farming could feasibly help support represent a ‘land sharing’ approach to nature conservation: cows grazing at low densities in herb-rich grasslands, interspersed with certain well-connected landscape elements, could provide habitat for a substantial diversity of wild species. However, such landscapes have diminished globally because of a focus on dairy production efficiency, with dairy farmers primarily paid for milk sold in a competitive global market where their contribution to biodiversity receives little reward. The Dutch Sustainable Dairy Chain (DuurzameZuivelKeten, or DZK)—a collaboration between key public and private dairy organisations in the Netherlands—has recently shown interest in calculating net biodiversity outcomes associated with national dairy production; and understanding to what degree the sector could support nature positive goals.

In defining nature positive, the Nature Positive Initiative states “Companies and financial institutions can contribute to the Nature Positive goal by taking these high-level actions: Assess their material impacts, dependencies, risks and opportunities; shift their business strategy and models; commit to science-based targets for nature; report their nature-related issues to investors and other stakeholders; transform by avoiding and reducing negative impacts, restoring, and regenerating nature; collaborate across land, seascapes and river basins; and advocate to governments for policy ambition”. A key point here is the concept of *contributing* towards an overarching absolute nature positive objective (say, on the scale of the Netherlands); which DZK could begin to do by first assessing impacts, and then developing a positive net outcome strategy within the production sector itself (see refs. ^8,10,17,18^). Note also that a nature positive pathway is considered by the NPI to be one in which connected global challenges beyond biodiversity (climate change, water use, other forms of environmental pollution—alongside other sustainability issues, such as human wellbeing) are solved in tandem; but here, we focus on biodiversity. Similarly to multiple other organisations, DZK has sought in the first instance to track net biodiversity outcomes against a strategy using one main focal biodiversity metric, acting as a proxy.

**Fig. 1:**
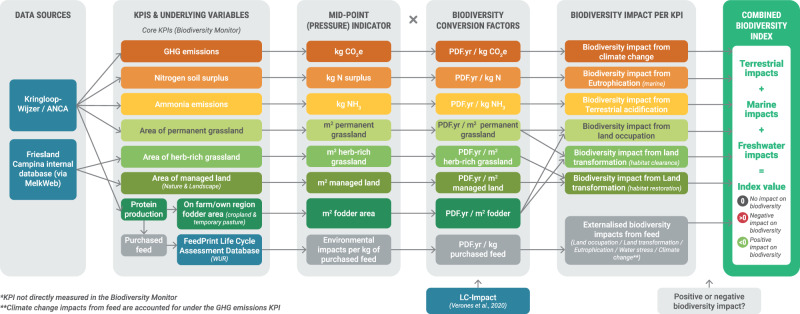
calculation of the integrated biodiversity index. A flow diagram showing the calculation of the specific biodiversity indicator we use here to track net biodiversity outcomes. Process incorporates biodiversity monitor key performance indicator data into a single aggregated index, measured in ‘PDF.year’. PDF potentially disappeared fraction.

Our analyses are underpinned by data collected using the Dutch Biodiversity Monitor. The Biodiversity Monitor for Dairy Farming, developed by FrieslandCampina, Rabobank and WWF (World-Wide Fund for Nature) Netherlands, aims to enable Dutch dairy farms to improve biodiversity outcomes whilst ensuring sustainable revenue. It uses seven key performance indicators (KPIs) to measure the environmental impacts of individual dairy farms (both positive and negative). The Biodiversity Monitor consequently provides the basis of one potential system for calculating losses and gains of biodiversity towards a net positive impact; by combining KPI data treated as environmental pressures, characterising those pressures into biodiversity impacts in terms of the potentially disappeared fraction (PDF) of species, and aggregating these into a single measure of PDF.year (Fig. 1; see the “Methods” section); i.e. the fraction of global species richness that could be lost if the relevant pressure persisted.

Here—having developed an integrated biodiversity index to act as a metric for monitoring progress towards a nature positive goal, we: (a) calculate overall sectoral impacts for a baseline year; (b) define a set of biodiversity ‘safeguards’, i.e. sub-targets to be achieved to ensure that a net positive outcome measured using the integrated index would not lead to perverse outcomes (e.g., by exceeding physical limits linked to one or more planetary boundaries); and (c) discuss broad strategies that could take the sector from baseline impacts towards positive net outcomes for biodiversity.

## Results

### Baseline annual biodiversity impacts

**Fig. 2:**
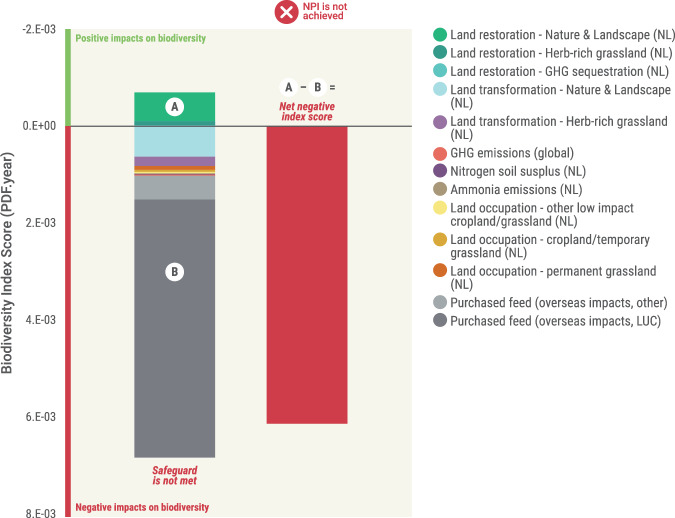
Baseline biodiversity impacts. Annual biodiversity impacts for the year 2020, in –PDF.year, broken down per KPI. LUC land use change, NL impacts within the Netherlands. *Note*: Other embedded impacts (non-LUC impacts) from purchased feed include those from land occupation, marine and freshwater eutrophication, and water consumption. Embedded greenhouse gas emissions associated with feed are included under the GHG emissions KPI. NPI net positive impact.

The baseline biodiversity impacts calculated for Dutch dairy production were the annual impacts for the year 2020, measured in terms of PDF.year (where PDF is the potentially disappeared fraction of species; Fig. 2). The KPIs from which the PDF-year estimates are derived (Fig. 1) come from a screened dataset consisting of 8950 farms (see the “Methods” section), which was then factored up to represent the whole sector—a total of 14,542 farms covering 870,880 ha^19^. The largest source of biodiversity impacts under our analysis comes from land transformation—mostly linked to the production of ingredients used in purchased feeds (e.g., concentrates and other roughage and by-products). Ingredients derived from oil palm products were linked to ~60% of land use change impacts associated with purchased concentrate feed (noting that land transformation impacts from soy, one of the key ingredients, were assumed to be zero, due to the sector currently sourcing 100% RTRS or equivalent certified soy). The second highest source of impacts was associated with land use change on farms within the Netherlands (negative impacts from land transformation and positive impacts from land restoration). This was calculated from annual reductions and increases in ecologically valuable landscape components (“Nature & Landscape”) and areas of herb-rich grassland: for example, a farm that reduced/increased its area of Nature & Landscape elements relative to the previous year would incur an associated biodiversity loss/gain. These losses and gains were then summed across farms to obtain the sectoral values.

Impacts from other KPIs are comparatively lower than those from land-related KPIs, which is consistent with land use change being the primary driver of global biodiversity loss^20^. However, this does not negate the importance of other KPIs (e.g., nitrogen soil surplus, ammonia emissions) which are particularly important as drivers of local to national biodiversity loss, and highly contentious issues within the Netherlands. It is commonly advised not to focus on the results for biodiversity footprints in PDF.year or similar metrics in absolute terms, but on the relative impacts across activities, which is more instructive^9^.

### Biodiversity safeguards

Though the practice is not uncommon, there are challenges in seeking to achieve a biodiversity net outcome measured using a single composite index. Not only are there considerable uncertainties in organisational biodiversity proxies (including those based on LCIA); there is also the potential to mask undesirable outcomes. For example, good performance against the index overall could reflect excellent performance across six KPIs, and unacceptable performance against the seventh. Consequently, we identified a set of safeguards specific to the Dutch dairy case to be implemented in conjunction with the biodiversity index; seeking to avoid perverse outcomes, and allowing biodiversity strategy to be set at the level of midpoint pressures rather than on the basis of the biodiversity index.

In the context of this project, based on literature review and stakeholder consultation (see the “Methods” section), we define a ‘safeguard’ as: a standard put in place to ensure that a positive net outcome is achieved while limiting any unintended, undesirable, or perverse biodiversity outcomes (given that ‘net outcomes’ approaches have at times led to the latter^6^).

Table 1 lists identified values for each safeguard category (see the “Methods” section), defined at the sector level (i.e., not for individual farms), and categorised into two groups that apply at different points of the mitigation hierarchy (a widely used framework for the preferred sequence of actions to take in mitigating biodiversity impacts, see e.g. ref. ^3^):

**Table 1:** Values/approach for Dutch dairy sector-level biodiversity safeguards

Safeguard category	Basis for safeguard	Proposed sector-level safeguard value	Notes on calculation for sector-level safeguard
*Safeguards for impact prevention*			
1. Avoiding irreplaceable biodiversity losses	Two-thirds (2/3) reduction in the amount of soy and oil palm products used relative to 2018 levels. Based on advice from the Committee on Land-based Dairy Farming (‘Advies Commissie grondgebondenheid’)	Less than or equal to ~150 million kg of soybean meal/flakes, ~6 million kg soy shells/hulls, and ~140 million kg of palm kernels.	Values are approximations calculated by using the total annual quantity (million kg of product) per feed type for soy and oil palm products listed in Table 8 of the Commissie Grondgebondenheid report^46^, which gives average values for 2016, based on a total of 1.7 million cows. We therefore scale these values to the number of cows in 2018 (~1.6 million according to Duurzame Zuivelketen^47^), as this is stated as the baseline year for comparison, and reduce by a third to calculate the safeguard value. Soy and palm oil are not necessarily the only possible safeguard to employ here, but are arguably those for which the most information is available.
	100% use of responsible soy (RTRS or equivalent) and use of responsible palm kernels in animal feed (RSPO or equivalent). Responsibly sourced soy and palm products are assumed to incur no impacts from deforestation (land use change). Based on the DZK goal for 2030.	100% of soy and oil palm products in feed certified as RTRS/RSPO or equivalent.	Note that a ‘book & claim’ method is currently used when buying responsible soy, although one option would be instead to shift to a ‘mass balance’ balance approach (including working with dairy sectors across Europe). It is also worth noting that dairy companies setting Science Based Targets for FLAG greenhouse gas emissions are also required to publicly commit to zero deforestation^48^.
2. Biophysical safeguards (here, the Biodiversity Monitor KPIs) Using van Doorn et al. (2019) as a basis)	KPI1: Percentage of permanent grassland (>5 years no tillage) Based on the CAP-GAEC (Common Agricultural Policy- Good Agricultural and Environmental Conditions)	>62%	In van Doorn et al.^49^, it is noted that nationally, the percentage of permanent grassland of the total area on dairy farms in 2017 was 62% (based on RVO data; noting that according to the RVO grassland becomes permanent grassland in the 6th year after 5 years of no tillage). This is used as the baseline from which permanent grassland should not decline. At the farm-level, van Doorn et al. recommended limits depending on the soil type (60%, 75%, and 80% for sandy, clay and peat soils, respectively). However, it is the overall sector value that is relevant here.
	KPI2: Percentage of protein from own land/region (<20 km) Based on the Advice Committee on Land-Relatedness (‘Advies Commissie grondgebondenheid’)	>65%	The DZK 2030 goal is for at least 65% of the protein in the cow’s ration must come from own land or within the local region of the dairy farmer. This is in order to reduce reliance on feed imported from other regions (linear nutrient flow) and stimulate greater proportions of grassland.
	KPI3: Nitrogen soil surplus Based on the Nitrates Directive Water Quality Standards (target values are based on Water Framework Directive Ecological Standards)	If based on human health standards of the Nitrates Directive: <105,000 tonnes of N soil surplus If based on ecological standards of the Water Framework Directive: <8700–35,000 tonnes of N soil surplus (depending on soil type breakdown)	van Doorn et al.^49^ calculated an average N soil surplus value for farms in locations where the Nitrates standard (50 mg NO_3_/l) was being met. This average value was equal to 120 kg N/ha, which (as our focus is the sectoral level) we assume that we can multiply by the total area of land use for Dutch dairy in 2020 (870,880 ha^19^) to give an order of magnitude indication for the safeguard value. A more detailed estimate could be achieved in future by calculating a weighted total based on soil and crop type (both of which influence the amount of N leached into water bodies). NB: the value in van Doorn et al.^49^ is based on the nationally transposed human health standards of the EU Nitrates Directive. We note that a safeguard value for biodiversity might ideally be determined on the basis of ecological standards (such as those as set out in the Water Framework Directive, at a value of 10–40 kg N/ha) but recognise that this would set a very stringent safeguard for Nitrogen soil surplus.
	KPI4: Ammonia emissions Based on the National Emissions Ceiling Directive (NECD) & Netherlands PAS Agreement (‘programmatic approach to Nitrogen’); although we note that new policy is under development.	<44,000 tonnes of NH_3_	van Doorn et al.^49^ calculated a sector-level value for ammonia emissions based on the NEC ceiling (21% reduction compared to 2005 levels) plus an additional 5600 tonnes NH_3_ reduction based on the PAS Agreements. This was equal to 44,000 tonnes NH_3_. A similar value (41,000 tonnes NH_3_) was arrived at by Beldman et al.^50^, based on a national 26% reduction target for Nitrogen deposition. We understand there is some debate as to whether this value is sufficiently low to safeguard ecosystems against NH_3_ emissions, but use it in the absence of an alternative and documented quantitative value.
	KPI5: Greenhouse gas emissions Based on the Forest, Land, and Agriculture (FLAG) Science Based Target Setting Draft Guidance^48^: General pathway for FLAG sectors (see Table 9 of the draft guidance).	35% reduction relative to 2020 levels	van Doorn et al.^49^ give a value of 12 million tonnes of CO_2_ equivalents as the sectoral limit for greenhouse gas emissions, based on dairy targets in the Netherlands Climate Agreement (Klimaatakkoord). However, this value is limited in scope to on-farm methane emissions within the Netherlands (from enteric fermentation and manure management). In order to account for the global scope of emissions from Dutch dairy and align with a 1.5 °C scenario, a science-based target approach is preferred. The proposed 35% reduction in absolute CO_2_ equivalents serves as a starting point, based on the general sector-level recommendations in the Forest, Land and Agriculture (FLAG) guidance published by SBTi^48^ (Table 9). This would need to be further elaborated for all scopes of emissions through setting detailed Science Based Targets for the sector; and we note also that the SBTi continues to develop and update new guidance regularly, with major releases on the SBTs linked to biodiversity coming out in 2023 onwards.
	KPI6: Percentage of herb-rich grassland Based on the ANLb (Agricultural Nature and Landscape Management)—preconditions for meadow bird habitat quality	15–20%	Here we apply the same percentage range as the farm-level value provided in van Doorn et al.^49^. This was calculated based on habitat requirements for meadow birds (the black-tailed godwit). The assumption is made that, outside of areas with habitat potential for black-tailed godwits (~67,000 ha—or ~8% of total dairy land in 2020—based on Melman and Sierdsema^51^), the 15–20% value would have similar benefits for other species groups (e.g., pollinators, other meadow bird species, soil biota, etc.). It also recognises that herb-rich grassland inherently increases the diversity of plant species (relative to more intensive grasslands and cropland). However, though making this assumption is the best option currently available, this safeguard should be updated through further research so that the evidence base explicitly extends to other species groups and regions. Relevant research is ongoing (coordinated by the Louis Bolk Institute), with results pending.
	KPI7: Percentage of Nature & Landscape elements Based on Cormont et al.^52^	7–10%	Same percentage as the average farm-level provided in van Doorn et al.^49^, based on research by Cormont et al.^52^ on the relationship between non-productive area and species richness on farmland. 7–10% of the total farm area is the value at which a ~50–60% increase in species richness is predicted, relative to a situation with no natural elements (noting that there is a lot of variation around this value).
*Safeguards for impact compensation*			
3. Geographic/spatial safeguards	Biodiversity gains (e.g., habitat restoration) should be located close to where impacts are occurring	Preliminary data analysis on the location of impacts for 2020 indicates that ~10–30% of biodiversity gains should be achieved in the Netherlands, and ~70–90% should be achieved overseas in regions where feed is sourced	This safeguard would apply to biodiversity gains being implemented to restore or offset any unavoidable impacts on biodiversity from Dutch dairy. The values would be updated each year and would be determined by the extent of unavoidable biodiversity impacts per region. For example, preliminary analysis of biodiversity impacts from Dutch dairy suggests that ~15% of impacts occurred within the Netherlands in 2020, and the remaining ~85% of impacts occurred overseas in locations where feed is sourced. Applying the geographic safeguard would mean that the same proportions should apply to biodiversity gains being implemented to compensate for these impacts. Note: These percentages apply to the unit used for the biodiversity index (‘PDF.year’); because of the way these values are calculated (e.g., including weighting for species vulnerability) these percentages would not directly translate into percentages of habitat restoration area. A given biodiversity gain (as measured using the biodiversity index score) could be achieved by restoring different absolute areas of habitat, depending on the geographical location.
4. Habitat type safeguards to ensure ecological equivalence	Biodiversity gains should be ecologically equivalent to biodiversity losses in order to achieve NPI.	Values determined year on year, based on specific impact areas.	Related to geographic safeguards, this safeguard would apply to biodiversity gains being implemented to restore/offset unavoidable impacts on biodiversity. The value(s) would be updated each year (dynamic) and would be determined by the extent of unavoidable biodiversity impacts per broad habitat type. For example, if, in a particular year, 20% of biodiversity losses occurred within wetland habitats, then 20% of biodiversity gains should also be within wetland habitats. In reality, the KPIs of the Biodiversity Monitor and associated biodiversity index (Stage 1) are too high-level to determine the exact types of habitat that would need to be compensated, so this may need to be based on average habitat types per region or require additional monitoring.
5. Temporal safeguards	Time lags between biodiversity losses and gains should be avoided.	Implementation of compensation should ideally be provided in advance (e.g., through biodiversity banking) or commence at the same time as impacts accrue. However, if there is some delay between ecological impacts and compensation, a multiplier will be added to gains requirements (as per Laitila et al.^23^). In any case, due to the practical feasibility around multiplier size, it is recommended that any time lag is no >5 years.	See Laitila et al.^23^, as well as the discussion on multipliers by Bull et al.^53^. We incorporate further consideration of this into the document for Stage 3.
	Ensure longevity of biodiversity gains	Are long-term management/monitoring plans in place for biodiversity gains? (Categorical: Yes or No).	Long-term in this case could be taken as ‘equal to or greater than one typical generation’, and certainly for the time frame for which the DZK strategy is to be defined i.e., ~ 30 years. Long-term management/monitoring of offset gains is a key offset design principle (see e.g. ref. ^21^). Further, one proposal is for DZK to align NPI targets with the CBD post-2020 strategy, seeking net gain for around 2050; which would mean monitoring for at least ~30 years.

#### Safeguards for impact prevention

Safeguards (1) and (2) define the minimum impacts that should first be avoided or reduced (i.e., the ‘unacceptable’ impacts on biodiversity). These are determined from ‘static’ goals or standards—such as the goal to buy 100% responsibly sourced soy, or to adhere to nitrogen standards as set out in the EU Nitrates Directive.

#### Safeguards for impact compensation

Safeguards (3)–(5) define how unavoidable impacts should be compensated in line with best practices for biodiversity (e.g., through restoring or offsetting). These are determined by the type of impacts that have taken place (e.g., the habitat types and locations that have been affected), and when those impacts occurred.

### Baseline values for sector-level KPIs

**Fig. 3:**
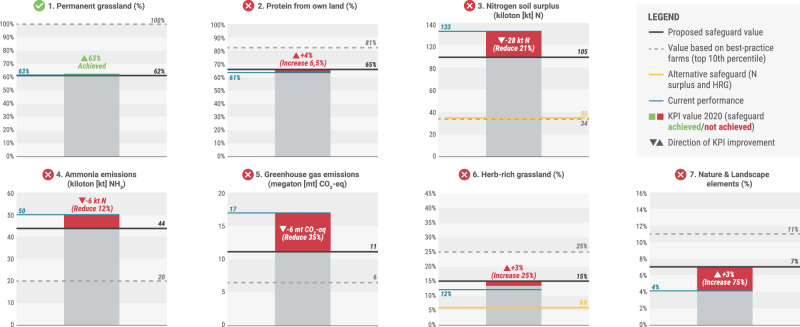
Performance by safeguard. Progress against proposed safeguards for 2020, with a range of scenarios included for comparison. Best practice = top 10th percentile; worst practice = <50th percentile. herb-rich grassland (HRG) and Nature and Landscape KPIs have high levels of uncertainty having been extrapolated from fewer data points. While the change in herb-rich grassland between 2019 and 2020 matches well with the ‘Natural Grassland’ figures reported by CBS for the dairy sector, our estimated total value of HRG is about three times larger than the Natural Grassland value reported by CBS for 2020.

Given the potential importance of the identified safeguards in developing a net positive strategy, we calculated the baseline values for current sector performance against each Biodiversity Monitor KPI separately (Fig. 3).

**Fig. 4:**
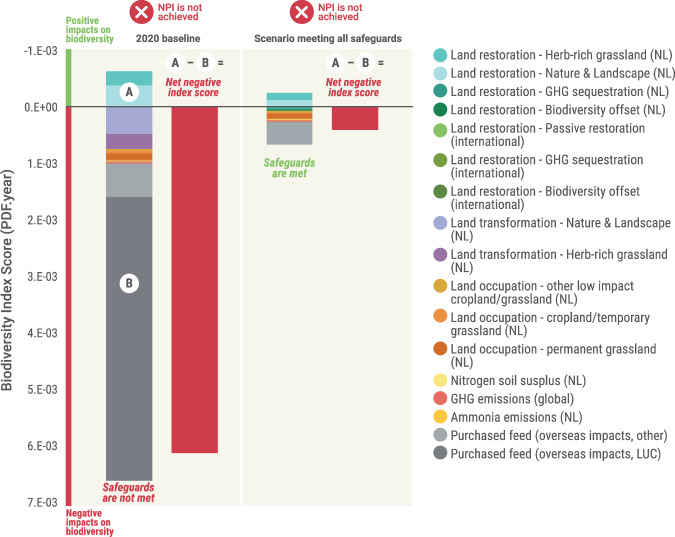
Biodiversity outcomes if all safeguards were met. Comparison between 2020 impacts vs. impacts if all proposed preventative safeguards were met at the sectoral level.

If all biophysical safeguards listed in Table 1 were met, the annual impact would be substantially less—but still, positive net outcomes (labelled for this specific project by DZK as net positive impact or NPI) would not be achieved without additional offsetting efforts (Fig. 4).

## Discussion

Our finding that a main contributor to overall sectoral biodiversity impacts is land use change in the supply chain is not necessarily unexpected (see refs. ^8,10^). However, the degree to which it dominates the biodiversity impact risks is; obscuring the importance of nitrogen surplus and ammonia emissions (as environmental, social, and political concerns) in the Netherlands. Thus, if any strategy was optimised using this index without safeguards as we proposed, it would run the risk of ignoring the importance of nutrient balances as a key issue in the Netherlands specifically. Biodiversity impacts can be considered at global, national, or local scales, and be assigned markedly different values across these scales—necessitating the inclusion of safeguards partly derived from local perspectives when performing calculations based on headline indicators and global trade flows. The safeguards employed here are specific to the Dutch case study, but the safeguard *categories* (Table 1) are not; the same categories could be used to structure safeguards for other agricultural production systems or even other sectors entirely. In essence, these categories draw from decades of work into biodiversity impacts mitigation hierarchies, extended to organisational or sectoral scales (see refs. ^2–4,9^).

Safeguards are likely especially important when one considers the degree of variability in results that can be obtained under the application of slightly different LCIA methods to calculate biodiversity footprints^21^; as without safeguards, any net biodiversity strategy developed based on that footprint would be highly sensitive to the specific LCIA route chosen. But targets for biodiversity *could* be set around the mid-point environmental pressures used in the LCIA process—in this case, the KPIs reported under the Biodiversity Monitor, which form part of the set of safeguards—as they are more precisely calculated and understood. In the meantime, the calculated endpoint biodiversity outcomes remain a useful tool for headline tracking of progress towards the desired net outcomes. Such an approach would be entirely consistent with current thinking on biodiversity impact calculation and disclosure, as recommended by, e.g. the Taskforce on Nature-Related Financial Disclosures^22^ or equally by policies such as the EU Corporate Sustainability Reporting Directive; which themselves are explicitly aligned with guidance on biodiversity target setting as per the Science Based Targets Network. Interestingly, meeting the safeguards alone in this case would potentially take dairy production in the Netherlands a long way towards fully mitigating biodiversity impacts (Fig. 4). It would also be a mechanism for ensuring alignment with other relevant existing policies and standards, at EU level and beyond (see detail in Table 1).

**Fig. 5:**
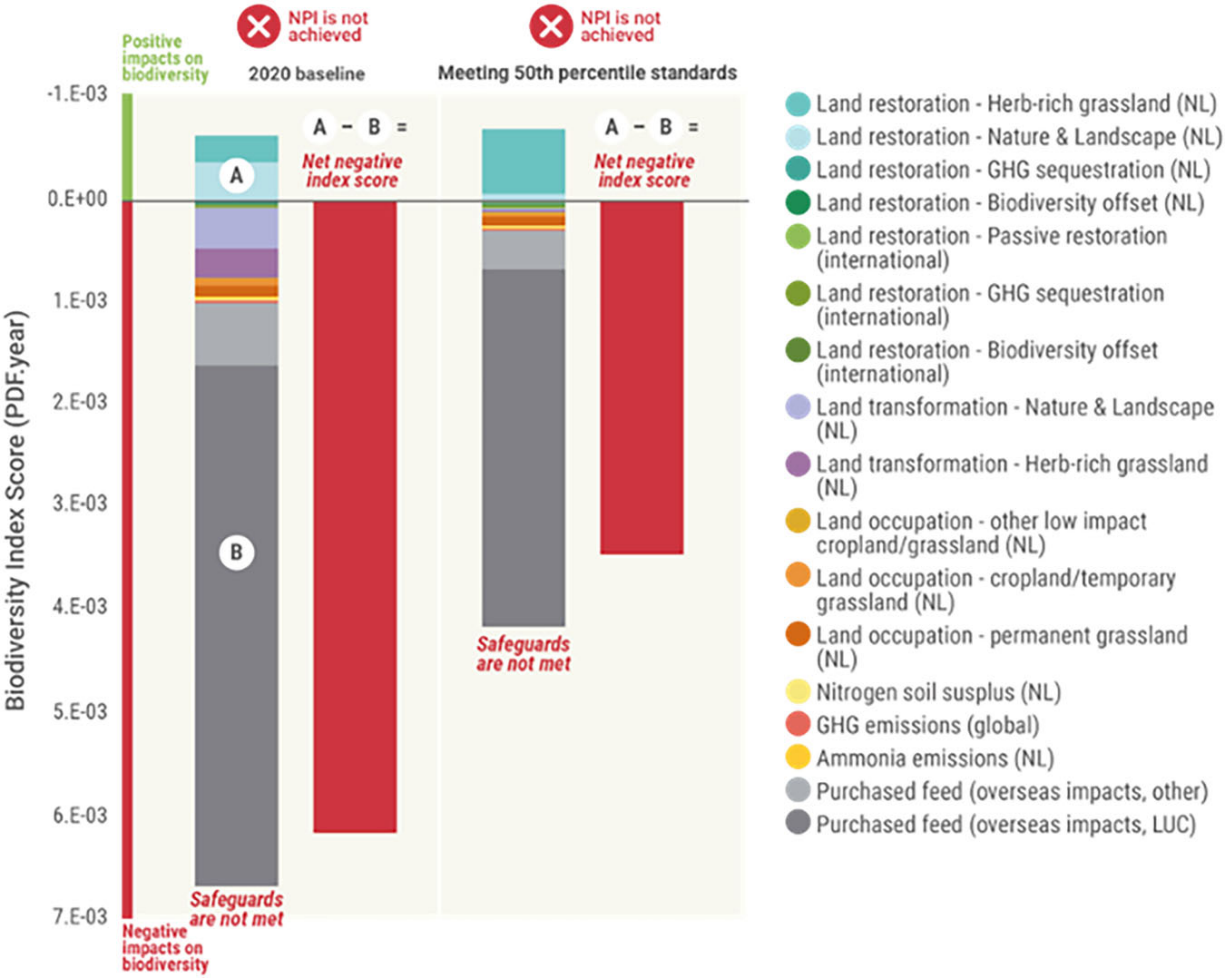
Biodiversity outcomes based on variable performance improvement. Comparison between 2020 impacts vs. impacts if farms below current 50th percentile standards on the index improved performance to current 50th percentile levels, based on the integrated index scores.

Importantly, beyond the likelihood that the use of safeguards would help avoid perverse outcomes for biodiversity, there will be other red lines from the perspective of the Dutch dairy sector associated with ‘mission critical’ (and consequently unavoidable) impacts on biodiversity (see ref. ^9^). In particular, since there is a requirement for milk production to continue, there will remain some inevitable negative impacts on biodiversity, which will require ecological compensation (such as biodiversity restoration offsets). The overall amount of dairy produced by the sector every year is a fundamental consideration, and whether dairy production is to increase, remain stable, or decrease year on year. The types of scenarios displayed in Fig. 4 should be interpreted in that context. For instance, if we apply linear regression to farm-level data, we find that: kg milk produced per hectare trends downwards with increasing % protein produced on farms’ own land (coefficient = −0.6), and trends upwards with increasing purchased concentrates per ha (coefficient = 0.9). It is important not to over-interpret these relationships, as they do not imply causation, and a full statistical treatment of the data would require far more nuanced analyses which is beyond scope here. However, such relationships would align with the reasonable contention that reducing biodiversity impacts via limiting feed imported from overseas will either require intensification of feed production within the Netherlands to maintain production or an overall reduction in milk production. So if the goal was to maintain dairy production while reducing the amount of feed purchased from overseas, there would be a requirement to give more land over to protein production within the Netherlands, implying less space to implement restoration of herb-rich grasslands and/or Nature and Landscape components to support domestic biodiversity gain. More extensive farms tend to be slightly larger on average, which may imply (given the positive relationship between purchased feed and production) that farm intensity rather than size plays a more substantial role in maintaining higher levels of production. In the case where the distribution of impacts by farm shifted to substantially lower impacts—such that farms in the current 90th percentile for impacts met current 50th percentile standards on those KPIs—we estimate that the result would be a 48% reduction in net impacts (in PDF.year) relative to 2020 values (Fig. 5). That might be considered more of an ‘even contribution’ approach towards net positive biodiversity outcomes, in which lower-performing farms were required to meet current ‘average’ performance. However, extrapolating based on correlations between KPI scores and production, this would also result in a ~26% reduction in milk production (assuming the same efficiency in processes and number of farms). Such nuances as those discussed in this paragraph, and the trade-offs associated with them, demonstrate the need to couch discussions around biodiversity metrics and relevant safeguards within a broader framework for sustainable development, which requires more detailed investigation than we have the space to perform here. Considerations, though, would certainly include those around the socio-economic implications of any given strategy for farmers nationally, as well as the implications internationally; particularly, for instance, in those regions that currently produce feed that is then exported to the Netherlands.

Considering all components of the analyses reported here, what possible pathways exist for the Dutch dairy sector to contribute towards nature positive i.e. the Global Biodiversity Framework post-2020 mission? We lay out three broad hypothetical pathways, with different implications for the sector, and therefore varying feasibility (although, arguably, with each option sequentially showing increasing alignment with global targets under the Global Biodiversity Framework). These are:

1. *‘Adaptive compensation’ approach*: In the case in which the mid-point KPI safeguards are seen as aspirational rather than strict ‘red-line’ limits, a scenario could involve making incremental progress year on year towards meeting all safeguards at the sector level—with all remaining annual impacts (initially close to baseline 2020 levels; Fig. 2) offset through restoration measures in the Netherlands and overseas. The offset activity required would be large but could be determined year on year via the methods here. Offsets would ideally commence in the same year as the impacts for which they compensate, with any delays leading to the use of multipliers (see ref. ^23^). Under this category of approach, it would be recommended to evaluate progress at milestones towards 2030 and on to 2050, to determine (a) the degree of feasibility of meeting all KPI safeguards, and (b) the practicality and cost of eventually implementing sufficient biodiversity offsets to compensate residual impacts.
  The approach is a ‘compensation’ (i.e. offsetting) focused approach (see ref. ^9^), with small initial efforts at impact prevention that increase steadily over time. It is a more feasible option in terms of the challenges of maintaining dairy production and of meeting safeguards. But this option could be costly in terms of financing biodiversity offsets requirements, where in any case offsetting should be a less preferred option on the spectrum of the mitigation hierarchy.
2. *‘Even contribution’ approach*: In this case, the initial focus would be on working with all farms in the lower-ranked 50% (index performance), seeking improvements that bring them up to the equivalent index score as the current 50th percentile (Fig. 5). Since these calculations are based on the integrated index score, and not on separate KPIs, they inherently incorporate some of the key interactions between KPIs. The result would be extensive prevention of biodiversity impacts for those farms, whilst those in the upper ranked 50% (again, by index score) would either continue business as usual or voluntarily seek improvements. The residual impacts would be compensated through biodiversity offsets, which would likely be far less extensive than in pathway 1 above. However, this is predicated on maintaining the number and area of existing farms; and the result of that (alongside achieving a drop in biodiversity impacts) would be a commensurate drop in dairy production. Our analyses—in the absence of any improved efficiencies in dairy production (which might allow greater production levels)—indicate a possible drop in dairy production of ~26%. The approach combines biodiversity impact prevention and compensation. However, it makes abundantly clear that there are inevitable trade-offs between biodiversity restoration, land availability for the dairy sector, and dairy production.
3. *‘Deep net positive’ approach*: This approach can be considered a maximum impact prevention approach. In one version of this case, all safeguards would be met at the sector-level (broadly assuming trade-offs at farm-level can be at least partly avoided), reducing impacts substantially (by ~94% according to our calculations; Fig. 4) and requiring minimal offsetting. In itself, this latter finding—that meeting basic biophysical and social safeguards (as outlined in Table 1) could proportionally deliver much of the progress needed towards neutralising biodiversity impacts—is worth underlining, and again emphasizes the usefulness of designing strategies based on multiple midpoint pressures and incorporating safeguards. Aside from the necessary changes in dairy production volumes, the deep net positive approach is appealing as it is likely not the case that a ‘one size fits all’ approach can or should be taken to applying the KPIs safeguards to farms. What this type of scenario does usefully do is illustrate the extent of the change needed if the sector were to take a primarily ‘avoidance focused’ approach towards meeting the net positive biodiversity target.

While considering pathways towards net positive outcomes, it is important to consider the issue of uncertainty in calculating impacts in the first place. The uncertainties involved in estimating biodiversity impacts using LCIA approaches—though considered the best available current practice—are numerous, and well documented^24^. Analyses specifically pertaining to the biodiversity impacts of dairy organisations based in the Netherlands have shown that even the *relative* impacts of different organisational activities can vary depending on the precise methodology employed^21^. This means in turn that nature strategies building on biodiversity footprints must be designed to account for uncertainty in the underlying approaches. It is not possible to make quantitative estimates of the uncertainty bounds involved, which remains a key research question for nature-positive efforts more generally^7,24^; rather, net positive strategies will likely have to be designed flexibly and with adaptive management practices built in.

Further, the safeguards outlined here were constructed around a combination of policy and literature review alongside non-systematic regional expert stakeholder consultation (see the “Methods” section). In principle, this provided a defensible basis for developing safeguards for our purposes. However, we note that the full application of a safeguards approach in practice would require a more detailed and nuanced stakeholder analysis, that is more widely built upon local knowledge, and systematically includes representatives of key stakeholder groups (see e.g. ref. ^10^).

The implementation of the Biodiversity Monitor has enabled us to perform a quantitative estimate of the Dutch dairy sector’s biodiversity impacts; here, we have done so based on established methodologies for Life Cycle Impact Assessment. This study shows one path for developing an integrated biodiversity index for a sector, and strategically developing key areas for targeting biodiversity impact reduction (e.g. land use change linked to purchased feed). However, our key conclusion here is that the strategy must also be guided by efforts to meet a suite of safeguards—both biophysical and social—which we identified in this case through literature review and stakeholder consultation. Otherwise, the use of the single index to guide strategy might result in certain important impacts sources being overlooked (for example, if certain impacts were estimated to be less substantial using the index methodology in question than they were under another methodology^21^), or some other perverse outcome (e.g. there is considerable scope for perverse outcomes when implementing compensatory measures like biodiversity offsets^19,23,25^). Furthermore, given the limitations inherent in any biodiversity metrics, we suggest that actual nature strategies tracked using LCIA-type metrics might be usefully structured around the midpoint environmental pressures exerted on nature. This is even more important when considering that biodiversity is only one aspect of nature recovery, and of sustainable development more broadly. Consequently, any biodiversity metric used to track progress will necessarily sit within a much wider monitoring framework featuring multiple dimensions of sustainability; GHG emissions, water consumption, and beyond.

The use of strategic safeguards linked to midpoint pressures, alongside an overarching index for tracking biodiversity outcomes, ends up providing a clear path forward for any biodiversity strategy. Meeting the safeguards alone would clearly take the sector a long way toward neutral net biodiversity outcomes. These conclusions are derived in relation to our case study but are likely generalisable to many other systems, in agricultural sectors and beyond: most sectors will have to construct indices for monitoring trends in net biodiversity outcomes if they are to track progress towards the Global Biodiversity Framework. Indeed, there are many regions with higher intact biodiversity than the Netherlands, for which balancing conservation and agricultural production is perhaps even more urgent. All countries have targets for biodiversity conservation alongside agricultural production under the Framework (e.g. Target 10 on enhancing sustainability in agriculture, Target 16 on enabling sustainable consumption), which approaches such as that taken here could support. For the reasons given here, organisations, sectors, and even nation-states should consider developing biodiversity strategies through mid-point pressures, and systematically develop safeguards alongside the key biodiversity metrics employed.

## Methods

### Biodiversity impact calculations

Our focal biodiversity metric is based on LCIA approaches, as is current good practice for comprehensive biodiversity footprinting (see e.g. ref. ^7^). The metric was based upon the KPIs monitored via the Biodiversity Monitor (see ‘Introduction’). Not only do these KPIs represent key pathways by which environmental pressures could impact biodiversity (e.g. GHG emissions, eutrophication); but they have also been agreed as relevant for DZK through a lengthy process of stakeholder engagement, and crucially, reflect data that are collected and therefore available to track long-term progress towards net biodiversity outcomes. To create a biodiversity index that integrated the KPI data (steps visualised in Fig. 1), conceptually, we:

1. Gathered available KPI data (measures of environmental pressure) for the seven KPIs described in the Biodiversity Monitor, for all dairy farms in the Netherlands. Note that there are at least three additional possible KPIs not currently included in the Biodiversity Monitor which the authorship team felt could be relevant for incorporation into future iterations of the Biodiversity Monitor, but for which data are not currently available. These are pesticide use, water consumption, and phosphorus soil surplus;
2. Treating each KPI as a ‘mid-point’ environmental pressure^10^, converted each KPI value into an estimate of biodiversity impacts using characterisation factors from the LC-Impact methodology. This was done at the level of KPIs for each individual farm—fully anonymised—to then be aggregated into a biodiversity impact per farm. Again, this is measured using the unit ‘Potentially Disappeared Fraction’ of species over time (PDF.year), which should be interpreted as an indicator for proportional contribution to global species extinction risk^25^. Here, PDF.year is used as a proxy indicator for combining and comparing biodiversity impacts across a range of mid-point pressures/KPIs; and,
3. Summed PDF.year values across all farms to provide an estimate of biodiversity impacts over a given year for dairy production in the Netherlands.

We include the seven KPIs described in the Biodiversity Monitor: GHG emissions, nitrogen soil surplus, ammonia emissions, area of permanent pasture, area of herb-rich grassland, area of managed land (labelled ‘Nature & Landscape’), and ‘protein produced on own land/in farmer’s own region’ (Table 2). The raw data underpinning the KPIs was used e.g. the actual area of different land use types, CO_2_(e) value for GHG emissions, etc. (Fig. 1). ‘Protein produced on own land/in farmer’s own region’ is an indicator of the level of self-sufficiency (e.g., feed produced on own land), but is consequently indicative of the environmental footprint in other parts of the world (e.g., to grow ingredients like soy, used in concentrate feeds). To calculate the environmental impacts associated with purchased feed, we make use of the FeedPrint NL database developed by Wageningen University & Research, Blonk Consultants, and GFLI. KPIs were assumed to qualitatively correlate either positively or negatively with biodiversity impacts (e.g. the greater the emissions of GHGs, the more negative the impacts on biodiversity = negative correlation; Table 2).

**Table 2:** List of KPIs, with their potential for generating biodiversity gains

KPI derived from the Biodiversity Monitor	KPI definition	Assumed relationship with biodiversity (+/−)	Further detail
GHG emissions (kg CO_2_e)	Total emissions of CO_2_, CH_4_, and N_2_O from ‘cradle to gate’ (i.e., the entire supply chain up to and including the dairy farm)	**–**	The greater the GHG emissions, the more negative the impacts on biodiversity. If GHG emissions were reduced to zero, then we treated associated biodiversity impacts as zero. We did not consider the possibility of sequestration (i.e. negative GHG emissions) in our model.
Nitrogen soil surplus (kg N surplus)	N supply per cultivation type minus N removal (crops) and emissions to air.	**–**	Impacts from N surpluses are localised (e.g., compared to CO_2_). There is either a surplus (negative impact) or no surplus (zero impact). Again, we assume no ‘sequestering’ additional N surplus to achieve biodiversity gains.
Ammonia emissions (kg NH_3_)	Total emissions of ammonia from the barn, manure storage, grazing, fertilisation using animal manure, use of fertiliser.	**–**	We assume that increased ammonia emissions mean more negative biodiversity impacts. Further, as with N or P soil surplus, NH_3_ emissions could be reduced to zero, but not become negative (i.e. be extracted from the environment)
Area permanent pasture (grassland) (ha)	Total area of permanent grassland—defined as a plot of grassland not included in the farm’s crop rotation for a minimum of 5 years.	**+/−**	We consider that an increase in permanent grassland would represent a biodiversity gain compared to cropland or temporary pasture, but could still be considered productive land with small impacts from ongoing occupation (e.g., by maintaining a lower diversity of grass species with some grazing pressure). Conversely, conversion of permanent grassland to herb-rich grassland or to ‘nature & landscape’ would be considered to represent an absolute biodiversity gain.
Area herb-rich grassland (ha)	Total area of herb-rich grassland (permanent grassland with a mix of at least 8 types of grass and herbs, often >10 types), including both extensive and productive herb-rich grassland, weighted according to its biodiversity value^54^.	**+**	Increase in herb-rich grassland can be interpreted as gain of ‘biodiversity rich’ grassland (noting that the KPI captures diverse types of herb-rich grassland which are already weighted in terms of their value for biodiversity). Therefore we assume a positive correlation.
Area Nature & Landscape (ha)	Total area of managed land under different nature & landscape packages/elements, weighted for its biodiversity value^54^.	**+**	Increase in the area managed or set aside for nature can be interpreted as an increase in biodiversity.
Protein/feed production (purchased feed (kg) and feed produced on own land/in own region (ha))	Purchased feed: Total quantity and type of purchased feed (concentrates, roughage, by-products). Feed from own land/region: Total area of land on own farm used to produce feed (e.g., fodder crops, grassland, etc.)	**+/−**	A reduction in land used to produce feed in the Netherlands or overseas could be interpreted as biodiversity gain, if that released agricultural land was allowed to restore to natural habitat. This cannot necessarily be assumed unless the same dairy production levels could be maintained on less land. Other impacts—e.g., reduction of overseas eutrophication impacts, or improving circularity in N/P nutrient flows within the Netherlands could be reduced/improved to zero but not achieve biodiversity gains (NB: embedded GHGs from feed would be included in the GHG emissions KPI).

We note the following assumptions:

- though the KPIs for ‘Nature & Landscape’ and ‘herb-rich grassland’ represent a range of different land management regimes (e.g., meadow bird management, landscape management, soil management, etc.), we treat them uniformly;
- the biodiversity index is calculated by farm and then aggregated. We assume that biodiversity gains across all farms have equal weight, and do not, for example, consider any strategic spatial placement of biodiversity gains as part of core areas or corridors;
- characterisation factors in LC-Impact^24^ are based on models that predict biodiversity losses per functional unit in terms of global species extinctions. To estimate biodiversity gains associated with certain activities e.g., Nature & Landscape management, we assume it is meaningful to apply these factors in reverse. This is a necessary assumption since, as far as we are aware, there are no comparable methods that model biodiversity gains from such a broad range of activities. Not only does this introduce some further uncertainty, but also we acknowledge that there is a temporal aspect here (in that reversing losses of biodiversity would likely take longer than causing it).

### General data attributes

Data were available at farm-level for 14,686 farms in total, with values spanning the time period 2018–2020. Since 2020 was the only year for which comparable data were available across all of the KPIs collected under the Biodiversity Monitor, it was chosen as the baseline year. Data on the herb-rich grassland and Nature & Landscape KPIs were provided by Royal FrieslandCampina (RFC), with data on the remaining KPIs and contextual data provided from the KringloopWijzer (KLW) database. To convert KPI values into a biodiversity index value for the sector as a whole, we converted KPIs provided as relative values (e.g., kg CO_2_e per kg milk, or kg NH_3_ per ha) into total/absolute values per farm, by multiplying by either the total farm area or total milk production per farm. Datasets were matched based on unique farm identifiers, but all farms were anonymised.

Farms were excluded from the analysis if they did not report comprehensive data under the Biodiversity Monitor across KPIs, for land area, or on production. Similarly, farms were excluded if there were discrepancies between the total area reported for the farm, and for the different summed habitat components (e.g. for different grassland types across the KPIs). We note that there is a possibility that exclusion could lead to analytical biases in terms of the types of farms that had data gaps/mistakes, though this is not something we can assume. Since the relevant farms were those that by definition we could not calculate biodiversity impacts for, due to data gaps/mistakes, this is not something we analysed. Instead, we note this as a potential issue and an avenue for further research. After screening, the final dataset consisted of 8950 individual farms (62% of all dairy farms in the Netherlands, based on a total number of dairy farms recorded by CBS in 2020 of 14,542^19^).

For each farm, the total area of combined temporary grassland and cropland combined was estimated by subtracting the area of permanent grassland from the total area of the farm. Yard area was assumed to be minor, and so not considered in this analysis. Areas of grassland/cropland were then adjusted to account for areas provided for Nature & Landscape. These Nature & Landscape management areas provided in the RFC dataset were separated into four categories, based on the type of land the measure would be applied to (e.g., grassland, cropland, or landscape element—as per the Cumulatie and Grondgebruik table provided by Boerennatuur) and also based on the biodiversity weighting per management package, (provided in the Beheerpakketten Biodiversiteitsmonitor (BBM) documentation and associated appendices^26,27^). These categories are shown in Table 3. The distinction between these four categories was necessary for biodiversity index calculations, separating areas of low-intensity farming (which it is assumed would have a relatively low biodiversity impact compared to more intensive areas) from farmland habitats capable of generating absolute biodiversity gains. It was necessary to exclude some management packages from calculations in order to avoid double counting by area: specifically, BBM107 and BB107 (‘Bodemverbetering grasland met ruige mest’, ‘Bodemverbetering bouwland met ruige mest’, ‘Bodemverbetering met ruige mest’, ‘Chemie en kunstmestvrij land’) as they are applied in combination with other management packages.

**Table 3:** Categorisation of Nature & Landscape and herb-rich grassland areas

Category	Description/definition	Examples	Type of impact on biodiversity	Relevant environmental pressure
Herb-rich grassland	Packages under the Grassland Management (Graslandbeheer) category of the BBM^26,27^	Extensive herb-rich grasslands, herb-rich grassland borders, Botanical grassland, old grasslands (>20 years) with herbs, etc.	Absolute biodiversity gain	Land transformation and restoration
Nature & Landscape areas	Nature & Landscape packages with a BBM weighting of >1	Hedges, groves, coppice areas, ‘plas-dras’ (wet grassland habitat), orchards, etc.	Absolute biodiversity gain	Land transformation and restoration
Low-impact arable land	Nature & Landscape packages with a BBM weighting of <1, typically applied to arable land	Stubble areas (‘stoppelland’), arable land with nesting field birds, arable land with clutch management, etc.	Relative biodiversity gain compared to more intensive farming	Land occupation
Low-impact grassland	Nature & Landscape packages with a BBM weighting of <1, typically applied to grassland	Grassland with rest period, extensive grazing, controlling water levels for meadow birds, etc.	Relative biodiversity gain compared to more intensive farming	Land occupation

Since it was not always possible to determine from the dataset which land use types were overlapping (e.g., whether HRG was included under temporary or permanent grassland). The following assumptions were therefore made:

- Area of HRG was subtracted from the total area of permanent grassland; and
- Areas of low-impact grassland/arable land and Nature & Landscape areas were subtracted from the assumed arable/temporary grassland area. If these values exceeded the arable/temporary grassland area, the remainder was subtracted from permanent grassland areas.

While this involves making several assumptions, it was considered necessary and reasonable as a means to account for potential double counting of areas.

For the 2020 analysis, areas of land transformation/restoration were calculated using the change in area of herb-rich grassland or Nature & Landscape management per farm relative to the previous year (2019). This calculation was only made when data were available for both years, which was the case for 1237 farms for herb-rich grassland (14% of the dataset, 9% of all farms in the Netherlands), and 3753 farms for Nature & Landscape areas (42% of the dataset, 26% of all farms in the Netherlands).

### Estimating environmental pressures associated with purchased feed

To estimate biodiversity impacts associated with purchased feed, we converted data provided on quantities of feeds into an estimated level of environmental pressure using the life cycle analysis (LCA) database FeedPrint (written consent was provided by the owners for this project). FeedPrint is also referred to in the calculation guidance for the KLW, particularly when calculating GHG emissions associated with feed, so we use FeedPrint as a consistent source for estimating all environmental pressures. FeedPrint provides ingredient- and country-level breakdown for a broad range of dairy feeds, and calculates the associated environmental pressures using the PEFCR-Feed methodology. We exported environmental data for feed components per country, with default parameters selected.

Data were provided for feed produced on the farms, and feed purchased by the farms. Feeds included concentrates, other roughage & by-products, maize, grass silage, and milk powder. It was assumed that the impacts of all feeds ‘produced’ by the farm were already accounted for by the other environmental KPIs (areas of grassland, total area of farm, N/NH_3_/CO_2_e emissions associated with growing fodder, etc.), so ‘produced’ feeds were excluded. Next, we assumed that ‘purchased’ grass and maize silage were produced on other farms within the Dutch dairy sector, and so were also excluded to avoid double counting.

Purchased concentrates and other roughage and by-products, however, were assumed to require additional analysis (i.e., produced by other sectors within the Netherlands, and/or by other countries). The analysis of purchased feeds therefore focuses on these two types of composite feeds.

To estimate the component ingredients in concentrates we used ‘concentrate dairy standard’ as a reference product, taking the ingredient and country-level breakdown directly from the FeedPrint database. ‘Other roughage & by-products’ is however a much broader category of feed ingredients; to determine constituent ingredients in this category, we referred to data published by Blonk Consultants^28^, which provided an in-depth life cycle inventory for Dutch semi-skimmed milk and semi-mature cheese. We referred specifically to Tables 3–10 of this report (using the most recent dry matter estimates provided within FeedPrint). The country-level breakdown for each of the composite ingredients was then sourced from FeedPrint, and can be viewed directly within the FeedPrint database software. Environmental values per kilogram of ingredient per country were exported for land occupation, freshwater eutrophication, marine eutrophication, and water consumption. Carbon values from FeedPrint were not used in order to avoid double counting, as these were assumed to be accounted for under the GHG emissions KPI (an assumption that follows KLW guidance).

While the area of land *occupation* is provided directly in FeedPrint, the area of land *transformation* (also known as land use change; LUC) is not. However, FeedPrint does provide estimates of carbon emissions associated with LUC, calculated using the PAS2050 methodology. Therefore, to calculate the area of LUC, we reverse this calculation using the same factors (provided in Annex C of the PAS2050:2011 guidelines). These factors are in the form of tonnes CO_2_e per ha per year, and are provided at local level for a set of countries. For countries not included in this list, we used the continental average.

The area of LUC was calculated for all feed ingredients except for those derived from soybean products. Soy was assumed to have no impacts associated with LUC due to the dairy sector's requirement for sourcing 100% certified sustainable soy (RTRS or equivalent; see Table 1).

### Calculation of biodiversity impact

There are several biodiversity metrics currently available for use in impact analysis, though none are widely accepted as standard. LCIA is an approach supported by leading frameworks (e.g. ref. ^22^), has precedent in the literature (e.g. ref. ^29^), and allows us to incorporate the full scope of environmental pressures (KPIs), converting these KPI ‘mid-points’ (pressures) into an estimated aggregated end-point impact on biodiversity.

LC-Impact software was chosen here because it is one of the most recently developed LCIA methodologies (developed as part of an EU FP7 project, via a collaboration between 14 partners). It incorporates spatial differentiation for environmental impacts where relevant, as well as levels of species vulnerability and endemism—both of which are lacking to some degree in other LCIA methodologies.

LC-Impact provides a set of characterisation factors (CFs), which can be used to calculate an estimate of biodiversity impact (in PDF.year) per unit of environmental pressure – for example, PDF.year per kg CO_2_e emitted, or PDF.year per m^2^ of land occupation. These CFs are based on a set of models from the scientific corpus that link the KPI to biodiversity via a particular ‘impact pathway’ (e.g., climate change, eutrophication, acidification, habitat conversion, etc.). Here, we mainly used the core set of CFs, using the marginal CF in the case of terrestrial acidification impacts linked to ammonia emissions; marginal CFs calculate the biodiversity impact of an additional kilogram of ammonia (as opposed to the average effect of a kilogram of ammonia). KPIs for CO_2_e emissions and NH_3_ emissions were combined directly with the relevant CFs. For other KPIs, further adjustments were made as follows:

- Nitrogen soil surplus describes the balance between supply (e.g., from fertiliser, manure, fixation, etc.) and removal (e.g., via crops or emissions to air) of nitrogen compounds on farms. We ‘capped’ negative nitrogen soil surplus values at zero—assuming that net removals of nitrogen would be highly localised, and should not be accounted for when aggregating across farms—before combining values with the biodiversity CFs. We also note for completeness that the CFs for nitrogen model eutrophication are based on a commonly applied (e.g. ref. ^30^, assumption of nutrient limitation—that is: nitrogen is assumed to be the limiting nutrient in marine ecosystems and phosphorus is assumed to be the limiting nutrient in freshwater ecosystems. As such, the impact of nitrogen soil surplus is modelled in terms of eutrophication of marine systems (this takes into account a generic soil leaching fraction for the Netherlands and nutrient transport via river systems—see Verones et al.^31^ for more information) rather than of freshwater systems. This could lead to impacts from nitrogen soil surplus being underestimated in our metric—although there is evidence that phosphorus is a primary driver of freshwater eutrophication, including within the Netherlands (e.g. refs. ^32–34^), and that nitrogen limitation tends to be stronger in marine systems^35^. Furthermore, relatively low levels of phosphorus soil surplus are reported for Dutch dairy farms—which would translate into a biodiversity impact from freshwater eutrophication several orders of magnitude lower than other KPIs ( ~ 1.4 × 10^−08^ PDF.year, based on an average of 7 kg P_2_O_5_ recorded on the WUR Agro & Food portal). So, though we have used the best method available, we acknowledge that this is a simplified approach. There are high percentages of eutrophic freshwater bodies in the Netherlands^36^, which would ideally be improved by CFs that take into account site-specific nutrient limitation and synergistic effects of nitrogen and phosphorus^37,38^.
- For land aspects, instead of applying the relevant factors provided via LC-Impact (based on a 2015 analysis), we substitute these for CFs calculated by Chaudhary and Brooks^39^. According to the authors of these resources, the 2018 factors are more up-to-date and reliable and can be directly substituted into LC-Impact. This also allows differences between three levels of land use intensity (minimal, light, intense) to be accounted for; definitions for each of the land use categories can be found in the supplementary material of Chaudhary and Brooks (2018). CFs for ‘light use’ pasture were applied to areas of permanent grassland, whereas CFs for ‘minimal use’ cropland and pasture were applied to areas of low-impact grassland/arable land. However, since ‘low-impact arable/grassland’ encompasses a broad range of management approaches with varying benefits for biodiversity, we also apply BBM weightings (see https://biodiversiteitsmonitor.nl/certificatie.html).
- For herb-rich grassland or areas under Nature & Landscape measures: Any decrease in area was assumed to represent a loss of habitat, so we applied the CF for the Netherlands; conversely, the increase in area represented a gain, and we applied the CF in reverse. We acknowledge that using reversed CFs to estimate ‘biodiversity gains’ is not standard; however, it was necessary since, as far as we are aware, there are no comparable methods that model biodiversity gains from such a broad range of activities. As for low-impact arable/grassland (above), herb-rich grassland and different types of Nature & Landscape management have variable effects on biodiversity, which are reflected in the BBM weightings. To avoid these weightings overestimating biodiversity gains (the maximum possible proportional change in habitat restoration is 1 i.e., 100% habitat recovery), the weightings were normalised such that the maximum possible weighting was 1. Though this means that we effectively assume complete restoration for these areas, which would not happen in reality on short timescales, this is likely within the large uncertainty bounds for applying LCIA to biodiversity footprinting in any case^24^.
- Areas undergoing LUC would also be associated with a change in GHG emissions; emissions associated with habitat loss, or sequestration through habitat restoration. We excluded GHG emissions from LUC linked to feed production, as those were already included within the values provided as part of the KLW dataset. Then, losses of habitat area were assumed to lead to an increase in GHG emissions, calculated using the values provided in Annex C of the PAS2050:2011 guidance. For any gains in habitat area (e.g., increases in herb-rich grassland or Nature and Landscape areas), GHG sequestration was estimated using the values provided by Table 3 of Schmidinger and Stehfest^40^, who calculate the average potential carbon sink per continental region and food product and provide factors in kg CO_2_/m^2^/year. GHG emissions and sequestration were both combined with the relevant CF in LC-Impact.

### *Accounting for other possible positive impacts*

#### Passive restoration

Agricultural land released from productive use could, in the absence of action by other sectors, passively restore to natural habitat over time (e.g. ref. ^41^). In general, however, land released from dairy production was not assumed to deliver a biodiversity gain via passive restoration as it would likely be used for other purposes (i.e. outside of the dairy sector). This is consistent with the literature on the treatment of counterfactual scenarios in net outcomes policies for biodiversity (e.g. ref. ^18^). However, there could be cases in which land was set aside but still retained by dairy sector actors, and in that case, any passive restoration should be included in the overall calculations. Therefore, passive restoration of released land is included in our model in the specific scenario where total milk production remains the same or increases, alongside a reduction in land use. In such cases, we apply a high temporal risk multiplier to reflect the uncertainty in restoration timescales (see below).

Biodiversity offsets are measurable conservation outcomes (e.g., restoring species and habitats) that are widely used to compensate for residual negative impacts on biodiversity. We did not include offsets in our models here, as these are not currently a component of DZK strategy. However, offsetting could be included in future versions of our models—and we note that biodiversity gains linked to biodiversity restoration offsets would likely be needed as part of achieving net positive impact overall.

### Applying multipliers to account for temporal risk for biodiversity gains

Time lags are important in conservation^42^. Impacts such as land use change may result in immediate biodiversity losses, but ecological gains from compensatory restoration activities may take time to accrue. Time lags are undesirable—particularly if species/habitats are threatened, or when the existence of biodiversity provides some ongoing ecosystem service that is diminished during the time lag.

A common approach is to apply a multiplier to areas being restored, in order to account for uncertainties around immediate/certain losses being compensated by delayed/uncertain future gains. The multiplier can be considered a ratio between damaged and necessary compensated amounts of biodiversity. It would be applied here as a factor to biodiversity gains, to calculate gains that account for these temporal uncertainties.

Laitila et al.^23^ propose a method to calculate minimum temporal multipliers associated with biodiversity restoration for offsets, which we apply to the biodiversity gains shown in Fig. 4 in the main text. Their method is based on the following parameters:

Time taken to restore different habitats (in number of years); The change in habitat condition (e.g., the proportional increase in biodiversity that is achieved by the restoration activity); A discount rate, which mathematically determines the currently perceived value of biodiversity gains that are not achieved until future years (‘net present value’); and, Permanence of the positive and negative impacts.

The values we applied for each parameter, for different restoration activities, are described in Table 4. The ‘permanence of impacts’ parameter was assumed to be 30 years for all habitats, being approximately equivalent to one generation (and therefore a feasible period of time for maintaining activities). Time lag values for habitat restoration are as advised by Meli et al.^41^ and via habitat management guidance provided by BoerenNatuur. Discount rates are as suggested by Overton et al.^43^. To calculate the change/improvement in habitat conditions associated with Nature & Landscape measures, we used the existing biodiversity weightings developed for the BBM and ANLb packages, which again were normalised as previously discussed.

**Table 4:** Parameters chosen for each of the three broad types of biodiversity restoration, and the final temporal multipliers calculated based on the tool/method provided by Laitila et al.^23^

Type of biodiversity restoration activity	Values chosen for parameters			Final multiplier based on Laitila et al.^23^
	1. Time to sufficient habitat restoration (years)	2. Proportional change in habitat condition	3. Discount rate (%)	
Conversion of land to Nature & Landscape management, or herb-rich grassland	10	BBM transitions for Nature & Landscape/herb-rich grassland	4	1.48
Active habitat restoration through biodiversity offsets	18	1 (assumes full habitat recovery)	4	2.02
Passive restoration of released agricultural land	35	1 (assumes full habitat recovery)	10	28.10

### Biodiversity impact estimates for the full dairy production sector

After characterising all KPIs in terms of endpoint biodiversity impacts using the approaches described above, biodiversity impact values (in PDF.year) were summed across all farms (cf. Fig. 1). The total impact estimated for the filtered dataset was factored up to an estimate for the production sector as a whole, based on the total number of farms (14,542^19^), and total area under dairy production in 2020 (870,880 ha^19^).

### Safeguard development

Setting any ‘net outcomes’ objective requires in part that any unavoidable biodiversity impacts are adequately compensated for (e.g., through biodiversity offsets). But to specify to what extent different biodiversity losses are permittable as part of net outcomes policies—and also in the context of uncertainties in calculating overall biodiversity impacts, as discussed throughout—we propose accompanying metrics with safeguards. These can be based both on empirical biophysical limits (at the level of the KPIs), as well as socio-political values. For example, a safeguard might specify that achieving net positive impacts on biodiversity should not be based on a strategy that includes deforestation of pristine rainforest through the Dutch dairy sector’s feed supply chain, or one that leads to levels of nutrient pollution within the Netherlands that are unhealthy for humans or wildlife. Further, strategies for biodiversity can be set at the level of safeguards on the relatively certain midpoint pressures (here, the KPIs) on the environment, and then monitored through the indicative if less certain endpoint biodiversity metrics.

It is unlikely that values can be maximised for all Biodiversity Monitor KPIs. For example, there may be farm-level trade-offs between KPIs (such as between protein produced on own land and nitrogen soil surplus, if production intensity is increased) or local constraints that limit performance. Safeguards help to define the safe operating space for the sector as a whole, allowing farm-level constraints and trade-offs to be navigated while striving for a genuine Net Positive impact on biodiversity at the sector level. Safeguards therefore need to be comprehensive enough to prevent perverse outcomes for nature, but practical enough to ensure feasibility when designing strategies towards a net positive target.

In developing safeguards here, we drew upon existing good practice guidance around achieving ‘net outcomes’ goals (such as net positive impacts on biodiversity), and associated topics (e.g. biodiversity offsetting). This includes peer-reviewed articles (e.g. ref. ^6^), business and financial institution standards and guidance (e.g., IFC Performance Standard 6^44^, UK BNG Good Practice Principles), policy guidance (e.g., EU biodiversity strategy, Dutch Natuurpunten^45^), and stakeholder consultation sessions. These resources exhibit some common themes, which potentially serve as a good basis for determining categories of safeguards (Table 5).

**Table 5:** Proposed safeguard categories based on Net Positive Impact principles

Principles for achieving Net Positive Impact	Brief description	Proposed safeguard category for Net Positive Impact from Dutch dairy
Applying the mitigation hierarchy	Prevention (avoidance or reduction) of impacts on biodiversity should be prioritised before considering ecological compensation (restoring or offsetting biodiversity impacts). This is because prevention of impacts is the least risky approach for safeguarding biodiversity. Impacts on irreplaceable biodiversity should always be avoided.	1. *Avoiding irreplaceable losses*: For example, large impacts should be avoided in highly biodiverse regions—such as deforestation caused by soy or oil palm in feed. DZK have expressed a goal that: “In 2025, dairy farming will be land-based in accordance with the advice of the Land-based Dairy Farming Committee (Advies Commissie grondgebondenheid).” This advice includes a proposed reduction in imports of soy and palm products used in feed by two-thirds by 2025 relative to 2018. DZK also requires 100% responsibly sourced soy (RTRS or equivalent) and the use of responsible palm kernels in animal feed (RSPO or equivalent).
Setting biophysical limits for unacceptable impacts	Related to (1) above, limits should be defined for the relevant components of biodiversity beyond which compensation for negative impacts is not acceptable.	2. *Biophysical safeguards*: These limits would be *sector-level* thresholds for each of the Biodiversity Monitor KPIs. They could be established using the farm-level values calculated by van Doorn et al.^49^ as a starting point.
The spatial proximity principle	Biodiversity gains should be located close to where impacts are occurring (e.g., in the same province, region, country, or ecoregion), and ideally contribute to locally strategic nature networks. In turn, this means that a biodiversity gain in one region should not compensate for a biodiversity loss in other regions. This spatial element also concerns the benefits people get from nature: the same people who lose access to nature should ideally benefit from biodiversity gains (or be compensated in other ways).	3. *Geographic/spatial safeguards*: Unavoidable impacts within the Netherlands should ideally be compensated within the region where they occurred (e.g., by restoring habitats and contributing to the National Ecological Network). Similarly, unavoidable impacts in locations where feed is sourced from should ideally be compensated within those regions. This could mean setting a minimum proportion of biodiversity gains that need to be achieved within the Netherlands, or within countries where feed ingredients are sourced, based on observed impacts from the sector.
Ensuring ecological equivalence	Biodiversity losses and gains should be equivalent (or ‘in-kind’): Habitats that have been lost should be replaced ‘like-for-like’ or better (in terms of conservation value). For example, an area of forest that has been cleared might not be compensated for by creating grassland.	4. *Habitat type safeguards*: In the context of this project, impacts on aquatic habitats (e.g., from nutrient runoff or leaching) should not be compensated for by restoring terrestrial habitats (e.g., on-farm biodiversity). This may mean that aquatic impacts can only be reduced or avoided, but not restored or offset.
Ensuring ecological compensation is timely and lasts	Limit the length of any time lags between causing biodiversity losses and implementing biodiversity gains. Ensure that biodiversity gains are designed to be maintained for a minimum amount of time.	5. *Temporal safeguards*: This is all about the timing of restoration measures. E.g., minimising time lags by setting a maximum number of years after biodiversity losses have occurred during which compensation for those impacts can be initiated (e.g., <5 years after negative impacts occur). Require biodiversity restoration measures to last at least as long as the impacts they are compensating for, and ensure long-term habitat monitoring and management plans are in place.

During our research period, two stakeholder consultation meetings were organised. The objective of these consultations was not only to inform relevant stakeholders about the project but also to receive feedback on the proposed methodologies. The stakeholder consultation meetings took place virtually on December 6, 2021, and May 25, 2022 (running for 2 h in both cases). Where identified stakeholders were unable to attend the meeting, they were invited to provide feedback by email. Both consultations were hosted by the research team. Representatives from the following organisations participated in the stakeholder consultation:

- Duurzame Zuivelketen (DZK)
- IMAGEN
- LTO
- Staatsbosbeheer
- Stichting Biodiversiteitsmonitor
- Wageningen University & Research (WUR)
- WWF France
- WWF Netherlands (WNF)

The first consultation focused primarily upon the biodiversity metric, and the second consultation upon proposed safeguards. Separate documents outlining the status of methodologies were provided in advance of the meeting to attendees, and the research team presented research updates at each. Stakeholders asked questions, provided feedback, and made suggestions on how to improve the approach and methods. Finally, the project team hosted a focussed discussion about critical points where stakeholder input was essential to move forward with the project. All stakeholder inputs were recorded and evaluated, and incorporated into refinements to the methodology where relevant and appropriate.

## Data Availability

KLW data from individual farms were linked to Nature and Landscape data from the same farms, and anonymized before being sent to the researchers i.e. completely anonymized. The data themselves are owned by ZuivelNL. The data have not been made public, so the research team is not permitted to share the dataset. Further, we use the FeedPrint NL database developed by Wageningen University & Research, Blonk Consultants, and GFLI; and, supporting data sourced from Royal FrieslandCampina and the KringloopWijzer database; again, these datasets can be requested from the corresponding owner organisations. The GLOBIO database, used to source characterisation factors for the analysis, is in the public domain.
